# ERICA: prevalence of dyslipidemia in Brazilian adolescents

**DOI:** 10.1590/S01518-8787.2016050006723

**Published:** 2016-02-02

**Authors:** José Rocha Faria, Vivian Freitas Rezende Bento, Cristina Pellegrino Baena, Marcia Olandoski, Luis Gonzaga de Oliveira Gonçalves, Gabriela de Azevedo Abreu, Maria Cristina Caetano Kuschnir, Katia Vergetti Bloch

**Affiliations:** I Centro de Epidemiologia e Pesquisa Clínica. Escola de Medicina. Pontifícia Universidade Católica do Paraná. Curitiba, PR, Brasil; II Programa de Pós-Graduação em Ciências da Saúde. Escola de Medicina. Pontifícia Universidade Católica do Paraná. Curitiba, PR, Brasil; III Universidade Federal de Rondônia. Porto Velho, RO, Brasil; IV Programa de Pós-Graduação em Saúde Coletiva. Instituto de Medicina Social. Universidade do Estado do Rio de Janeiro. Rio de Janeiro, RJ, Brasil; V Faculdade de Ciência Médicas. Núcleo de Estudos da Saúde do Adolescente. Universidade do Estado do Rio de Janeiro. Rio de Janeiro, RJ, Brasil; VI Instituto de Estudos em Saúde Coletiva. Universidade Federal do Rio de Janeiro. Rio de Janeiro, RJ, Brasil

**Keywords:** Adolescent, Dyslipidemias, epidemiology, Prevalence, Cardiovascular Diseases, Cross-Sectional Studies

## Abstract

**OBJECTIVE:**

To determine the distribution of total cholesterol, LDL cholesterol, HDL cholesterol, and triglycerides in Brazilian adolescents, as well as the prevalence of altered levels of such parameters.

**METHODS:**

Data from the Study of Cardiovascular Risks in Adolescents (ERICA) were used. This is a country-wide, school-based cross-sectional study that evaluated 12 to 17-year old adolescents living in cities with over 100,000 inhabitants. The average and distribution of plasma levels of total cholesterol, LDL cholesterol, HDL cholesterol, and triglycerides were evaluated. Dyslipidemia was determined by levels of total cholesterol ≥ 170 mg/dl, LDL cholesterol ≥ 130 mg/dl, HDL cholesterol < 45 mg/dL, or triglycerides ≥ 130 mg/dl. The data were analyzed by gender, age, and regions in Brazil.

**RESULTS:**

We evaluated 38,069 adolescents – 59.9% of females, and 54.2% between 15 and 17 years. The average values found were: total cholesterol = 148.1 mg/dl (95%CI 147.1-149.1), HDL cholesterol = 47.3 mg/dl (95%CI 46.7-47.9), LDL cholesterol = 85.3 mg/dl (95%CI 84.5-86.1), and triglycerides = 77.8 mg/dl (95%CI 76.5-79.2). The female adolescents had higher average levels of total cholesterol, LDL cholesterol, and HDL cholesterol, without differences in the levels of triglycerides. We did not observe any significant differences between the average values among 12 to 14 and 15- to 17-year old adolescents. The most prevalent lipid alterations were low HDL cholesterol (46.8% [95%CI 44.8-48.9]), hypercholesterolemia (20.1% [95%CI 19.0-21.3]), and hypertriglyceridemia (7.8% [95%CI 7.1-8.6]). High LDL cholesterol was found in 3.5% (95%CI 3.2-4.0) of the adolescents. Prevalence of low HDL cholesterol was higher in Brazil’s North and Northeast regions.

**CONCLUSIONS:**

A significant proportion of Brazilian adolescents has alterations in their plasma lipids. The high prevalence of low HDL cholesterol and hypertriglyceridemia, especially in Brazil’s North and Northeast regions, must be analyzed in future studies, to support the creation of strategies for efficient interventions.

## INTRODUCTION

Cardiovascular diseases (CVD) are the main cause for morbidity and mortality in societies with Western lifestyles^25^. In Brazil, CVD is the main cause of death^24^. In addition of promoting great socioeconomic impacts to patients and their families, CVDs also imply high costs to the state, due to the high frequency of hospital admissions, medical leaves of absence, and early retirement. Such impact may be even higher in the next few years, as the mortality rate due to some forms of CVD is increasing in some of Brazilian regions^1^. Thus, controlling the risk factors for atherosclerotic disease, which are the pathophysiological base of coronary ischemic events and of a significant share of cerebrovascular ischemic events, is essential to change such scenario^28^.

The alterations of plasma lipids and their lipoproteins are associated with elevated cardiovascular risk^8^. Elevated cholesterol associated with low-density lipoprotein (LDLc) is closely correlated with increased cardiovascular risk, regardless of age^11^. Although the onset of atherothrombotic events usually takes place after the fourth decade of life, early exposure to hyperlipidemic environments may lead to lipid deposition on artery walls in the first weeks after birth^16^. Necropsy data reveal that high LDLc and low levels of cholesterol associated with high-density lipoprotein(HDLs) is associated with coronary atherosclerosis in adolescents and young adults^15^. Therefore, cardiovascular prevention must start in childhood and adolescence, and, to do that, it is necessary to identify the presence of risk factors in this population.

In Brazil, population-based data on alterations in plasma lipids are scarce and generally have restrict casuistry, small samples, or very limited geographical areas^19^ ^,^ ^23^. The aim of this analysis was to determine the distribution of total cholesterol (TC), LDLc, HDLc, and triglycerides (TG) in Brazilian adolescents, as well as the prevalence of altered levels of those parameters.

## METHODS

This work is part of the cross-sectional, nation-wide, school based Study of Cardiovascular Risks in Adolescents (ERICA) conducted in 2013-2014. The aim of ERICA was to estimate the prevalence of diabetes mellitus, obesity, cardiovascular risk factors, and markers of insulin resistance and inflammation in 12- to 17-year old adolescents attending schools in Brazilian cities with over 100,000 inhabitants.

The research sample was stratified in 32 strata comprising 27 capitals and five sets of municipalities with over 100,000 inhabitants in each of Brazil’s five geographical regions. For each geographical stratum, the larger schools were, the more chances they had of being selected. On the other hand, the farther away from a state capital a school was, the fewer chances it had of being picked. The sample is representative of the population of teenage students at national and regional levels, and for the Brazilian capitals. Parameters such as the location of a school (urban or rural) and management (public or private) were also considered. That strategy allowed to concentrate the sample in the surrounding areas of capitals, reducing costs and making the study logistics easier, especially regarding the collection of blood and the standardization of pre-analytical procedures. In total, 1,247 schools (out of 1,251 selected) were evaluated in 122 Brazilian municipalities (out of 124 selected). Further details on the sampling process are found in the publication by Vasconcellos et al.^27^

In the second sampling stage, we selected three groups from each school, considering combinations of sessions (morning and afternoon) and eligible grades (seventh, eighth, and ninth grade of elementary school and first, second, and third grade of high school). All students from selected groups were invited to take part in ERICA, but only morning session students took part in the blood collection as they were required to be fasting. Adolescents outside the age range between 12 and 17 years, pregnant ones, and physically or mentally-challenged adolescents (either temporarily or permanently) were excluded from the analyses for not being considered eligible.

The collected data were obtained through the use of self-administered questionnaires in handheld computers (PDA – personal digital assistant). The questionnaire had around 100 questions divided in 11 blocks: sociodemographic aspects, occupational activities, physical activity, diet habits, smoking, use of alcohol, reproductive health, oral health, sleeping and waking hours during the week and on weekends, physical morbidity (self-reported), and mental health. Also, data regarding weight, height, waist and arm perimeters, arterial pressure, and food intake – this one through the reported 24-hour. dietary recall. Details on the experiment were previously described^2^.

A standardized research protocol was adopted for blood collection and applied in the 27 centers. We used only a reference laboratory, concentrating all biochemical analyses of the study, with strict quality assurance and support from local partner laboratories that managed the collection and receiving of samples. This allowed for measures to be standardized and results to be made uniform. All laboratories received the instructions on the protocol to be followed in all steps, from scheduling to transportation to the central unit, including labeled kits for collecting blood of each adolescent. We instructed the adolescents to fast for 12 hours before the collection and applied a questionnaire before the exam to confirm students had fasted.

The exams conducted were: TG, HDLc, glucose, glycated hemoglobin, fasting insulin, and TC. LDLc was calculated by Friedewald’s formula^4^. Table 1 presents the methods used to analyze each exam and adopted cutoff points^28^. Dyslipidemia was defined upon the presence of high TC, LDLc, or TG levels, or low HDLc levels. The glucose parameters are not shown in this article.

**Table 1:** Methods for laboratory analysis and reference values. ERICA, Brazil, 2013-2014.

Lipid	Method^a^	Equipment	Reference values^b^		

			Desirable	Threshold	High
Triglycerides (mg/dL)	Enzyme kinetics	ADVIA 2400 Siemens	< 100	100-129	≥ 130
Cholesterol (mg/dL)	Enzyme kinetics	ADVIA 2400 Siemens	< 150	150-169	≥ 170
LDLc (mg/dL)	Friedewald Equation	(calculated)	< 100	100-129	≥ 130
HDLc (mg/dL)	Enzyme colorimetric assay	ADVIA 2400 Siemens	≥ 45	-	-

LDLc: low-density lipoprotein cholesterol; HDLc: high-density lipoprotein cholesterol

^a^ Sociedade Brasileira de Patologia (Brazilian Pathology Society).

^b^ V Brazilian Guidelines on Dyslipidemias and Prevention of Atherosclerosis, 2013.

This analysis included information on gender, age in years, and age range (12-14 and 15-17), type of school (public or private), and regions in Brazil (North, Northeast, Midwest, Southeast, and South).

Prevalences and 95% confidence intervals (95%CI) were calculated for each component in the lipid profile by gender, age, and type of school, covering the national and the regional context. Averages and proportions were described for the quantitative and categorical variables, respectively, with 95%CI in both cases.

The distribution of characteristics was adjusted according to the sampling design, by using complex statistical sampling routes, as the sample of ERICA employs stratification (each of the 27 capital cities and five strata with the set of municipalities with over 100,000 inhabitants from each of Brazil’s five regions) and clustering (by school and by group) in its selection stages. Sampling weights were calculated by the multiplication of the inverse inclusion probabilities in each stage of the sampling and calibrated by considering the projected number of adolescents enrolled in schools located in the geographical strata that were considered in December 31, 2013. A post-stratification estimator was used – it modifies the natural weight of the design through a calibration factor. That factor corresponds to the ratio between the total population and the total estimated by the natural weight of the design for the post-stratum or domain of estimation considered. Further details on the sampling design can be found in Vasconcellos et al^27^. The analyses were conducted in statistical software Stata^a^, version 14.0.

The study was approved by the Research Ethics Committees (REC) of the Central Coordination of the study (Institute of Collective Health Studies of the Universidade Federal do Rio de Janeiro) and of the institutions responsible for conducting the study in each Brazilian state. All adolescents interviewed and examined handed in informed consent terms signed by their legal guardians.

## RESULTS

We analyzed the data from 38,069 adolescents, who answered the questionnaire and had the lipid profiles measured in ERICA. The national coverage for blood collection was 52.7%. Table 2 describes the characteristics of the sample. Around two thirds of the adolescents were females, and most of them studied at public schools.

**Table 2:** Sample distribution by gender, age range, type of school (public or private), and geographical region. ERICA, Brazil, 2013-2014.

Variable	Sample	%
General	38,069	100
Gender		
Male	15,247	40.0
Female	22,822	60.0
Age		
12-14 years	17,434	45.8
15-17 years	20,635	54.2
Gender and vage		
Male, 12-14 years	7,140	18.8
Male, 15-17 years	8,107	21.3
Female, 12-14 years	10,294	27.0
Female, 15-17 years	12,528	32.9
School		
Public	28,167	74.0
Private	9,902	26.0
Region		
North	7,322	19.2
Northeast	11,821	31.1
Southeast	8,653	22.7
South	4,758	12.5
Midwest	5,515	14.5

**Figure 1:**
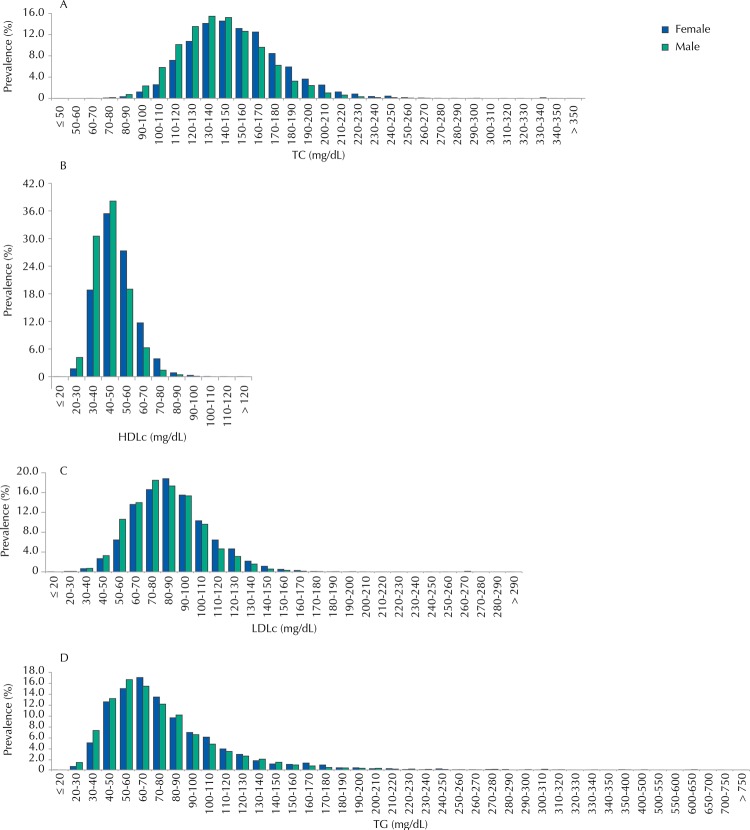
Distribution of blood levels TC, HDLc, LDLc, and TG in adolescents. ERICA, Brazil, 2013-2014.

TC: total cholesterol; HDLc: high-density lipoprotein cholesterol; LDLc: low-density lipoprotein cholesterol; TG: triglycerides

The distribution of lipid values by gender in the sample is presented in Figure 1, and the averages are shown in Table 3. Generally speaking, and considering the stratification by gender and age, the averages are very close to one another for all plasma lipids analyzed. The values from the female adolescents are always higher than the ones of male adolescents.

**Table 3:** Averages and 95%CI of plasma lipids, and prevalence and 95%CI of threshold values and values altered by gender and age range and estimated altered populationa,b. ERICA, Brazil, 2013-2014.

Lipids	Average		Threshold		High		Population estimated with alterations
		
	mg/dl	95%CI	%	95%CI	%	95%CI	
Total cholesterol							
All	148.1	147.1-149.1	24.2	22.7-25.8	20.1	19.0-21.3	2,940,705
Male	143.6	142.4-144.8	22.7	20.4-25.2	15.3	13.9-16.9	1,256,102
Female	152.6	151.4-153.9	25.7	24.5-27.0	24.9	23.4-26.5	1,684,602
12-14 years	149.4	148.0-150.7	25.8	24.3-27.4	20.7	19.1-22.5	937,793
15-17 years	147.1	145.8-148.3	22.8	20.8-24.9	19.6	18.0-21.2	2,002,911
LDLc							
All	85.3	84.5-86.1	19.5	18.5-20.5	3.5	3.2-4.0	1,526,733
Male	83.4	82.2-84.5	17.4	16.0-18.9	2.9	2.3-3.6	669,805
Female	87.2	86.3-88.1	21.5	20.2-22.9	4.3	3.7-4.9	856,928
12-14 years	86.2	85.1-87.3	20.6	19.0-22.4	3.7	3.1-4.4	467,877
15-17 years	84.5	83.5-85.5	18.4	17.2-19.7	3.4	2.9-4.1	1,058,856
Triglycerides							
All	77.8	76.5-79.2	12.0	11.0-13.0	7.8	7.1-8.6	1,312,329
Male	76.4	74.7-78.1	10.9	9.8-12.2	7.6	6.5-8.8	610,449
Female	79.3	77.8-80.7	13.0	11.8-14.2	8.1	7.3-9.0	701,880
12-14 years	78.9	76.7-81.0	12.7	11.0-14.6	8.3	7.2-9.5	434,638
15-17 years	76.9	75.8-78.1	11.3	10.2-12.4	7.4	6.6-8.4	877,690
			Low %				
HDLc							
All	47.3	46.7-47.9	46.8	44.8-48.9	-	-	3,104,161
Male	44.9	44.4-45.5	55.9	53.7-58.2	-	-	1,256,003
Female	49.6	48.9-50.3	37.8	35.4-40.2	-	-	1,848,158
12-14 years	47.4	46.7-48.1	45.0	42.3-47.8	-	-	819,980
15-17 years	47.2	46.4-48.0	48.4	45.9-50.8	-	-	2,284,181

LDLc: low-density lipoprotein cholesterol; HDLc: high-density lipoprotein cholesterol

^a^ alteration = higher threshold values.

^b^ The population estimates for the domains were obtained through the processing of microdata from IBGE’s Demographic Sensuses 2000 and 2010.

In the assessment of dyslipidemia prevalence, a higher percentage of female adolescents had high levels of TC and LDLc. On the other hand, the prevalence of low HDLc levels was lower for the female gender.

We observe no differences between genders regarding prevalence of hypertriglyceridemia and also no differences in the average lipid levels of the youngest adolescents (12 to 14 years old) as compared to the oldest ones (15 to 17 years). Likewise, the prevalences of dyslipidemias were not different between those groups.

**Figure 2:**
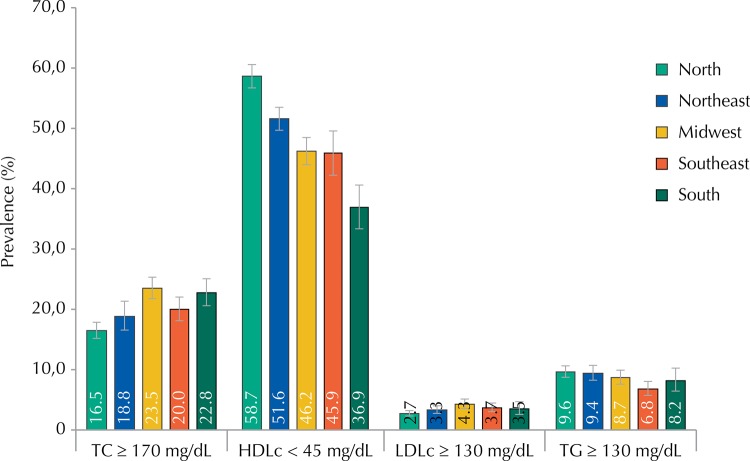
Prevalence of dyslipidemia according to geographical regions. ERICA, Brazil, 2013-2014.

TC: total cholesterol; HDLc: high-density lipoprotein cholesterol; LDLc: low-density lipoprotein cholesterol; TG: triglycerides; N: North; NE: Northeast; MW: Midwest; SE: Southeast; S: South

Figure 2 shows the prevalences of dyslipidemia according to Brazilian regions. No differences for TC, LDLc, and TG were observed among the five regions, with similar percentages of alterations. Prevalences of low HDL cholesterol were high in Brazil’s North and Northeast regions.

## DISCUSSION

Although measures for nationwide primary prevention may seem to be reducing some types of CVD in Brazil^21^, the economic impact of these diseases in our environment is significant. The development of effective strategies for cardiovascular prevention depends on the proper acknowledgment of its risk factors in the country. ERICA is the largest study on the prevalence of cardiovascular risk factors in adolescents ever conducted in Brazil. The prevalences of lipid alterations were high in the Brazilian adolescents who study in municipalities with over 100,000 inhabitants, especially the ones of low HDLc levels and high TC levels.

High cholesterol is correlated with cardiovascular risk^11^ and, even in children and adolescents, is associated with the presence of sub-clinical atherosclerosis^12^ and with the risk of dyslipidemia at adult ages^20^. In ERICA, the definition of altered values followed the references of a national guideline^28^ that differs from other proposals, such as the National Cholesterol Education Program (NCEP)^17^. These differences in the definitions of dyslipidemia must be considered when comparing the results from ERICA with other populations. Comparisons are even harder due to the scarcity of nationwide data in most countries. The current prevalence of dyslipidemia in children and adolescents in the United States and its time series between 1999 and 2012 have been recently published^10^. Around one fifth of American adolescents have some sort of lipid alteration, and this percentage decreased in the evaluated period. In ERICA, the same percentage is only found for TC. Nonetheless, a significantly higher proportion has low HDLc levels in Brazil. Although it involves a population of very distinct ethnicities, a nationwide study conducted in South Korea found average TC, LDLc, HDLc, and TG values similar to the ones found in our population^29^. In HELENA, a multicenter, European study with students at the same age range as ERICA, lower TG levels were observed as compared to the ones from ERICA (68 mg/dL [SD = 34]) for the whole population, with similar patterns of gender distribution: 63 mg/dL (SD = 31) in the male gender and 73 mg/dL (SD = 36) in the female gender^22^.

In turn, the National Health and Nutrition Examination Survey (NHANES) III (1988-1994) – a North-American study – found higher lipid levels than the ones of ERICA for all parameters, TC 163 mg/dL (standard error [SE] = 1.0); LDLc 95 mg/dL (SE = 1.6); HDLc 49 mg/dL (SE = 0.4), and TG 93 mg/dL (SE = 2.4)^6^.

The coverage and the national representativeness of ERICA study found never-before-seen data in our population. Distinct aspects of age ranges and genders were observed. No consensus exist on whether using single reference values – as used in ERICA and more widespread in the clinical routine – or using curves with specific values for gender and age is ideal for defining the presence of dyslipidemia in adolescents.

Magnussen et al.^13^ analyzed which strategy among the available ones would be best to predict the presence of dyslipidemia in adulthood, by using the reference values of NCEP^17^ and curves for gender and age originated from NHANES^7^. Although the evaluation through curves has allowed for better predicting low HDLc levels during adulthood, all the remaining lipid parameters were better predicted by the reference values of NCEP (similar to the ones used in ERICA). In ERICA, age was not a factor that determined significant differences. The average levels TC, LDLc, HDLc, and TG did not differ among 12-14 and 15-17-year old adolescents, corroborating the idea that single reference values for adolescents may be used to better identify lipid alterations^28^. That information reinforces the need for properly knowing the reference values used herein by the ones to provide medical care to such population, which is not observed nowadays^5^.

In this initial analysis, no specific curves using year-to-year ages were used, but the studies that used them showed slightly reduced TC, LDLc, and HDLc in the early adolescence of male subjects, whose levels returned to their previous values at the end of adolescence. On the other hand, in female adolescents, age does not seem to have a direct and rising relationship with LDLc levels^7^. This difference between genders became evident in ERICA*.* The TC, LDLc, and TG average values were higher in female adolescents, as well as the HDLc values. Consequently, the prevalence of high TC, LDLc, and TG levels were also higher in the female gender, which had the smallest prevalence of low HDLc.

In this study, we found regional differences regarding prevalence of dyslipidemias. Prevalence of low HDLc cholesterol was significantly higher in Brazil’s North and Northeast regions. Prevalence of high TG was also higher in those two regions, but confidence intervals were juxtaposed. In Recife, Northeast region, a study conducted with public school students also found high prevalence of low HDLc (56.0% [95%CI 51.3-60.5])^19^. The combination of those metabolic alterations, low HDLc, and high TG is usually present, especially in obese patients^3^ and patients with improper lifestyles. Together, those parameters are markers of the presence of smaller, denser, and more proatherogenic LDL molecules. That pattern of dyslipidemia, in which there is little alteration of LDL levels with predominance of HDLc and TG alterations, was already described as a predominant pattern during childhood^9^. Recent data of increased mortality due to cardiac ischemic diseases in those regions^1^ suggest that different regions in Brazil may be in distinct stages of the epidemiological transition process, which was already well described in different countries in the Americas^14^. Future analyses in the same population will allow for better understanding the factors associated with that pattern of atherogenic dyslipidemia that was observed in adolescents from Brazil’s North and Northeast regions.

Although it had been the least frequent alteration, the finding of 3.6% of high LDLc prevalence (≥ 130 mg/dl) deserves special attention. The presence of LDLc levels in children and adolescents must be the first step for acknowledging family hypercholesterolemia, a disease with genetic causes and autosomal dominant onset, which affects around 1 in 500 individuals. In Brazil, at least 1.0% of the population with family hypercholesterolemia is estimated to be correctly identified^18^. Adolescents suffering from that disease are exposed to a high lipid burden from birth, and need specialized medical supervision. Without proper treatment, around 50.0% of men will have a coronary event before the age of 50^26^. Therefore, the data from ERICA are a unique opportunity for evaluating the real prevalence of family hypercholesterolemia in Brazil. From the identification of index cases, planning a cascade screening strategy will be possible.

This initial analysis of the lipid data from ERICA aimed at describing the distributions of lipids and dyslipidemias in teenage students of Brazilian municipalities with over 100,000 inhabitants. Investigating factors associated with dyslipidemias and their combinations, such as food intake characteristics, lifestyle, and history of family morbidity may be enabled by future analyses. Although ERICA is a sectional study, as it deals with a population of adolescents who still do not have clinical complications or mortality due to dyslipidemias, it is unlikely that associations that may be observed result from survival bias.

In short, lipid alterations are frequent in Brazilian adolescents. Lifestyle interventions are fundamental to improve this scenario, and they are generally effective in the short run. The data suggest that, even though prevention strategies are planned countrywide, it is fundamental to recognize regional differences, so that these strategies can be properly executed.

## Footnotes

a Stata Corp. Stata: statistical software for professionals. Version 14.0. College Station, Texas; 2015.
