# Supplementary material for: ERICA: prevalence of dyslipidemia in Brazilian adolescents
Source: Rev Saude Publica. 2016 Feb 2;50(Suppl 1):10s. doi: 10.1590/S01518-8787.2016050006723 (PMC4767041; doi:10.1590/S01518-8787.2016050006723)
Supplement: Translation

# Translation: ERICA: prevalência de dislipidemia em adolescentes brasileiros

**Authors:** José Rocha Faria, Vivian Freitas Rezende Bento, Cristina Pellegrino Baena, Marcia Olandoski, Luis Gonzaga de Oliveira Gonçalves, Gabriela de Azevedo Abreu, Maria Cristina Caetano Kuschnir, Katia Vergetti Bloch

**Affiliations:** I Centro de Epidemiologia e Pesquisa Clínica. Escola de Medicina. Pontifícia Universidade Católica do Paraná. Curitiba, PR, Brasil; II Programa de Pós-Graduação em Ciências da Saúde. Escola de Medicina. Pontifícia Universidade Católica do Paraná. Curitiba, PR, Brasil; III Universidade Federal de Rondônia. Porto Velho, RO, Brasil; IV Programa de Pós-Graduação em Saúde Coletiva. Instituto de Medicina Social. Universidade do Estado do Rio de Janeiro. Rio de Janeiro, RJ, Brasil; V Faculdade de Ciência Médicas. Núcleo de Estudos da Saúde do Adolescente. Universidade do Estado do Rio de Janeiro. Rio de Janeiro, RJ, Brasil; VI Instituto de Estudos em Saúde Coletiva. Universidade Federal do Rio de Janeiro. Rio de Janeiro, RJ, Brasil

**Keywords:** Adolescente, Dislipidemias, epidemiologia, Prevalência, Doenças Cardiovasculares, Estudos Transversais

## Abstract

**OBJETIVO:**

Determinar a distribuição de colesterol total, LDL-colesterol, HDL-colesterol e triglicerídeos em adolescentes brasileiros, bem como a prevalência de níveis alterados de tais parâmetros.

**MÉTODOS:**

Foram utilizados dados do Estudo de Riscos Cardiovasculares em Adolescentes (ERICA), estudo transversal, de âmbito nacional e base escolar que avaliou adolescentes de 12 a 17 anos, residentes em municípios com mais de 100 mil habitantes. Foi avaliada a média e distribuição dos níveis plasmáticos de colesterol total, LDL-colesterol, HDL-colesterol e triglicerídeos. Dislipidemia foi determinada por níveis de colesterol total ≥ 170 mg/dl, LDL-colesterol ≥ 130 mg/dl, HDL-colesterol < 45 mg/dL ou triglicerídeos ≥ 130 mg/dl. Os dados foram analisados por sexo, idade e regiões do Brasil.

**RESULTADOS:**

Foram avaliados 38.069 adolescentes, 59,9% do sexo feminino; 54,2% com idade entre 15 a 17 anos. Os valores médios encontrados foram: colesterol total 148,1 mg/dl (IC95% 147,1-149,1), HDL-colesterol 47,3 mg/dl (IC95% 46,7-47,9), LDL-colesterol 85,3 mg/dl (IC95% 84,5-86,1), e triglicerídeos 77,8 mg/dl (IC95% 76,5-79,2). Adolescentes do sexo feminino apresentaram níveis médios de colesterol total, LDL-colesterol e HDL-colesterol mais elevados, mas sem diferença nos níveis de triglicerídeos. Não houve diferença significativa de valores médios entre adolescentes de 12 a 14 e de 15 a 17 anos. As alterações com maior prevalência foram HDL-colesterol baixo (46,8% [IC95% 44,8-48,9]), hipercolesterolemia (20,1% [IC95% 19,0-21,3]) e hipertrigliceridemia (7,8% [IC95% 7,1-8,6]). O LDL-colesterol elevado foi observado em 3,5% (IC95% 3,2-4,0) dos adolescentes. As prevalências de HDL-colesterol baixo foram mais elevadas nas regiões Norte e Nordeste do País.

**CONCLUSÕES:**

Uma parcela significativa dos adolescentes brasileiros apresenta alterações dos lípides plasmáticos. A alta prevalência de HDL-colesterol baixo e a hipertrigliceridemia, sobretudo nas regiões Norte e Nordeste do Brasil, devem ser analisadas em futuros estudos para subsidiar formulações de estratégias de intervenções eficazes.

## INTRODUÇÃO

As doenças cardiovasculares (DCV) são a principal causa de morbimortalidade nas sociedades de hábitos de vida ocidentais^25^. No Brasil, a DCV é a principal causa de morte^24^. Além de promoverem um grande impacto socioeconômico ao paciente e seu núcleo familiar, as DCV geram altos custos ao Estado, em decorrência da alta frequência de internações, licenças médicas e aposentadorias precoces. Este impacto poderá ser ainda maior nos próximos anos, pois a taxa de mortalidade por algumas formas de DCV está aumentando em algumas regiões do País^1^. Assim, o controle dos fatores de risco para a doença aterosclerótica, base fisiopatológica dos eventos isquêmicos coronarianos e de parcela significativa dos eventos isquêmicos cerebrovasculares, é de fundamental importância para mudança deste cenário^28^.

As alterações dos lípides plasmáticos e suas lipoproteínas estão associadas à elevação do risco cardiovascular^8^. Elevações do colesterol associado à lipoproteína de baixa densidade (LDLc) guardam estreita correlação com aumento do risco cardiovascular, independentemente da faixa etária^11^. Embora a manifestação clínica dos eventos aterotrombóticos ocorra habitualmente após a quarta década de vida, a exposição prematura a um ambiente hiperlipidêmico pode levar à deposição lipídica na parede arterial já nas primeiras semanas após a concepção^16^. Dados de autópsia revelam que LDLc alto e níveis reduzidos de colesterol associado à lipoproteína de alta densidade (HDLc) associam-se à aterosclerose coronariana em adolescentes e adultos jovens^15^. Portanto, medidas de prevenção cardiovascular devem ser iniciadas na infância e na adolescência, e para isso, é necessária a identificação da presença dos fatores de risco nessa população.

No Brasil, dados populacionais sobre as alterações dos lípides plasmáticos são escassos, em geral com casuísticas restritas, amostras pequenas ou áreas geográficas muito delimitadas^19^ ^,^ ^23^. O objetivo desta análise foi determinar a distribuição de colesterol total (CT), LDLc, HDLc e triglicerídeos (TG) nos adolescentes brasileiros, bem como a prevalência de níveis alterados desses parâmetros.

## MÉTODOS

Este trabalho faz parte do Estudo de Riscos Cardiovasculares em Adolescentes (ERICA), estudo transversal, nacional, de base escolar, conduzido em 2013-2014. O objetivo do ERICA foi estimar a prevalência de diabetes mellitus, obesidade, fatores de risco cardiovascular e de marcadores de resistência à insulina e inflamatórios em adolescentes de 12 a 17 anos que frequentam escolas em municípios brasileiros com mais de 100 mil habitantes.

A população da pesquisa foi estratificada em 32 estratos constituídos por 27 capitais e cinco conjuntos de municípios com mais de 100 mil habitantes em cada uma das cinco regiões geográficas do País. Para cada estrato geográfico, as escolas foram selecionadas com probabilidade proporcional ao tamanho e inversamente proporcional à distância da capital. A amostra é representativa da população de adolescentes escolares em âmbito nacional, regional e para as capitais. Questões como localização da escola (área urbana ou rural) e administração (pública ou privada) também foram consideradas. Essa estratégia permitiu concentrar a amostra no entorno das capitais, o que reduziu custos e facilitou a logística do estudo, principalmente em relação à coleta de sangue e adequação de procedimentos pré-analíticos. No total, foram avaliadas 1.247 escolas (de 1.251 selecionadas) em 122 municípios (dos 124 selecionados). Mais detalhes sobre o processo de amostragem encontram-se na publicação de Vasconcellos et al.^27^

No segundo estágio amostral, foram selecionadas três turmas de cada escola, considerando-se combinações de turno (manhã e tarde) e anos elegíveis (sétimo, oitavo e nono do ensino fundamental e primeiro, segundo e terceiro do ensino médio). Todos os alunos das turmas sorteadas foram convidados a participar do ERICA, mas apenas alunos do turno da manhã, devido à necessidade de jejum, participaram da coleta de sangue. Foram excluídos das análises, por não serem considerados elegíveis, os adolescentes que não pertenciam à faixa etária de 12 a 17 anos, adolescentes grávidas e os indivíduos com deficiência física ou mental, temporária ou permanente.

Os dados coletados foram obtidos usando-se questionário autopreenchível e aplicado com o uso de coletor eletrônico de dados, denominado PDA (*personal digital assistant*). O questionário continha cerca de 100 questões divididas em 11 blocos: aspectos sociodemográficos, atividades ocupacionais, atividade física, comportamento alimentar, tabagismo, uso de bebidas alcoólicas, saúde reprodutiva, saúde bucal, horas de dormir e acordar durante a semana e no final de semana, morbidade física (autorreferida) e saúde mental. Também foram coletados dados de peso, estatura, perímetros da cintura e do braço, de pressão arterial e de consumo alimentar, este último pelo recordatório alimentar de 24 horas. Detalhes sobre o protocolo do estudo foram previamente publicados^2^.

Para a coleta de sangue, foi adotado protocolo de pesquisa padronizado e aplicado nos 27 centros. Foi utilizado apenas um laboratório de referência, no qual foram centralizadas todas as análises bioquímicas do estudo, com rígido controle de qualidade, apoiado por laboratórios parceiros locais que gerenciaram a coleta e o recebimento de amostras, permitindo a padronização de medidas e a uniformidade dos resultados. Todos os laboratórios receberam documentação com o protocolo a ser seguido em todas as etapas, do agendamento ao transporte para a unidade central, incluindo o *kit* etiquetado para coleta de sangue de cada adolescente. Os adolescentes foram orientados a ficar em jejum por 12 horas antes da coleta. Um questionário foi aplicado antes do exame para confirmar o cumprimento do jejum.

Os exames realizados foram: TG, HDLc, glicose, hemoglobina glicada, insulina de jejum e CT. O LDLc foi calculado pela fórmula de Friedewald^4^. A Tabela 1 apresenta os métodos utilizados para análise de cada exame e os pontos de corte adotados^28^. Foi definido como dislipidemia a presença de níveis elevados de CT, LDLc ou TG, ou níveis baixos de HDLc. Os parâmetros glicêmicos não estão apresentados neste artigo.

**Tabela 1:** Métodos da análise laboratorial e valores de referência. ERICA, Brasil, 2013-2014.

Lipídeo	Método^a^	Equipamento	Valores de referência^b^		

			Desejável	Limítrofe	Elevado
Triglicerídeos (mg/dL)	Cinética enzimática	ADVIA 2400 Siemens	< 100	100-129	≥ 130
Colesterol (mg/dL)	Cinética enzimática	ADVIA 2400 Siemens	< 150	150-169	≥ 170
LDLc (mg/dL)	Equação de Friedewald	(calculado)	< 100	100-129	≥ 130
HDLc (mg/dL)	Ensaio colorimétrico enzimático	ADVIA 2400 Siemens	≥ 45	-	-

LDLc: colesterol associado à lipoproteína de baixa densidade; HDLc: colesterol associado à lipoproteína de alta densidade

^a^ Sociedade Brasileira de Patologia.

^b^ V *Brazilian Guidelines on Dyslipidemias and Prevention of Atherosclerosis*, 2013.

Na presente análise, foram utilizadas informações sobre: sexo, idade em anos e por grupo etário (12-14 e 15-17), tipo de escola (pública ou privada) e regiões do Brasil (Norte, Nordeste, Centro-Oeste, Sudeste e Sul).

Foram calculadas as prevalências e intervalos de confiança de 95% (IC95%) de cada componente do perfil lipídico por sexo, idade e tipo de escola, contemplando os âmbitos nacional e regional. Foram descritas médias e proporções para as variáveis quantitativas e categóricas, respectivamente, com IC95% em ambos os casos.

A distribuição das características foi ajustada segundo o delineamento amostral, utilizando-se rotinas estatísticas para amostragem complexa, visto que a amostra do ERICA emprega estratificação (cada um dos 27 municípios de capital e cinco estratos com o conjunto de municípios de mais de 100 mil habitantes de cada uma das cinco regiões do país) e conglomeração (por escola e por turma) em seus estágios de seleção. Os pesos amostrais foram calculados pelo produto dos inversos das probabilidades de inclusão em cada estágio da amostra e foram calibrados considerando a projeção do número de adolescentes matriculados em escolas localizadas nos estratos geográficos considerados em 31/12/2013. Foi utilizado um estimador de pós-estratificação, que modifica o peso natural do desenho por um fator de calibração. Esse fator corresponde à razão entre o total populacional e o total estimado pelo peso natural do desenho para o pós-estrato ou domínio de estimação considerado. Mais detalhes sobre o delineamento da amostra encontram-se em Vasconcellos et al.^27^ As análises foram realizadas no pacote estatístico Stata 14.0^a^.

O estudo foi aprovado pelos Comitês de Ética em Pesquisa (CEP) da instituição da Coordenação Central do estudo (Instituto de Estudos em Saúde Coletiva, da Universidade Federal do Rio de Janeiro) e das instituições responsáveis pela condução em cada estado brasileiro. Todos os adolescentes entrevistados e examinados forneceram o termo de consentimento livre e esclarecido assinado por seus responsáveis.

## RESULTADOS

Foram analisados os dados de 38.069 adolescentes que responderam ao questionário e tiveram a dosagem de perfil lipídico realizada no ERICA. A cobertura nacional para coleta de sangue foi de 52,7%. A Tabela 2 descreve as características da amostra. Cerca de 2/3 dos adolescentes eram do sexo feminino e a maioria, proveniente de escolas públicas.

**Tabela 2:** Distribuição da amostra por sexo, grupo etário, tipo de escola (pública ou privada) e região geográfica. ERICA, Brasil, 2013-2014.

Variável	Amostra	%
Geral	38.069	100
Sexo		
Masculino	15.247	40,0
Feminino	22.822	60,0
Idade		
12-14 anos	17.434	45,8
15-17 anos	20.635	54,2
Sexo e idade		
Masculino, 12-14 anos	7.140	18,8
Masculino, 15-17 anos	8.107	21,3
Feminino, 12-14 anos	10.294	27,0
Feminino, 15-17 anos	12.528	32,9
Escola		
Pública	28.167	74,0
Privada	9.902	26,0
Região		
Norte	7.322	19,2
Nordeste	11.821	31,1
Sudeste	8.653	22,7
Sul	4.758	12,5
Centro-Oeste	5.515	14,5

A distribuição dos valores de lípides na amostra, por sexo, está ilustrada na Figura 1 e as médias são apresentadas na Tabela 3. Observa-se que, de maneira geral e considerando a estratificação por sexo e idade, as médias são muito próximas entre si, para todos os lípides plasmáticos analisados. Os valores dos adolescentes do sexo feminino são sempre maiores do que os dos adolescentes do sexo masculino.

**Figura 1:** Distribuição dos níveis séricos de CT, HDLc, LDLc e TG nos adolescentes. ERICA, Brasil, 2013-2014.

CT: colesterol total; HDLc: colesterol associado à lipoproteína de alta densidade; LDLc: colesterol associado à lipoproteína de baixa densidade; TG: triglicerídeos

**Tabela 3:** Médias e IC95% dos lípides plasmáticos e prevalência e IC95% de valores limítrofes e alterados por sexo e faixa etária e população estimada com alteraçãoa,b. ERICA, Brasil, 2013-2014.

Lípides	Média		Limítrofe		Elevado		População estimada com alteração
		
	mg/dl	IC95%	%	IC95%	%	IC95%	
Colesterol total							
Todos	148,1	147,1-149,1	24,2	22,7-25,8	20,1	19,0-21,3	2.940.705
Masculino	143,6	142,4-144,8	22,7	20,4-25,2	15,3	13,9-16,9	1.256.102
Feminino	152,6	151,4-153,9	25,7	24,5-27,0	24,9	23,4-26,5	1.684.602
12-14 anos	149,4	148,0-150,7	25,8	24,3-27,4	20,7	19,1-22,5	937.793
15-17 anos	147,1	145,8-148,3	22,8	20,8-24,9	19,6	18,0-21,2	2.002.911
LDLc							
Todos	85,3	84,5-86,1	19,5	18,5-20,5	3,5	3,2-4,0	1.526.733
Masculino	83,4	82,2-84,5	17,4	16,0-18,9	2,9	2,3-3,6	669.805
Feminino	87,2	86,3-88,1	21,5	20,2-22,9	4,3	3,7-4,9	856.928
12-14 anos	86,2	85,1-87,3	20,6	19,0-22,4	3,7	3,1-4,4	467.877
15-17 anos	84,5	83,5-85,5	18,4	17,2-19,7	3,4	2,9-4,1	1058.856
Triglicerídeos							
Todos	77,8	76,5-79,2	12,0	11,0-13,0	7,8	7,1-8,6	1.312.329
Masculino	76,4	74,7-78,1	10,9	9,8-12,2	7,6	6,5-8,8	610.449
Feminino	79,3	77,8-80,7	13,0	11,8-14,2	8,1	7,3-9,0	701.880
12-14 anos	78,9	76,7-81,0	12,7	11,0-14,6	8,3	7,2-9,5	434.638
15-17 anos	76,9	75,8-78,1	11,3	10,2-12,4	7,4	6,6-8,4	877.690
			% Baixo				
HDLc							
Todos	47,3	46,7-47,9	46,8	44,8-48,9	-	-	3.104.161
Masculino	44,9	44,4-45,5	55,9	53,7-58,2	-	-	1.256.003
Feminino	49,6	48,9-50,3	37,8	35,4-40,2	-	-	1.848.158
12-14 anos	47,4	46,7-48,1	45,0	42,3-47,8	-	-	819.980
15-17 anos	47,2	46,4-48,0	48,4	45,9-50,8	-	-	2.284.181

LDLc: colesterol associado à lipoproteína de baixa densidade; HDLc: colesterol associado à lipoproteína de alta densidade

^a^ alteração = valores limítrofes + elevados.

^b^ As estimativas de população para os domínios foram obtidas por meio de processamento dos microdados dos Censos Demográficos de 2000 e 2010 do IBGE.

Na avaliação da prevalência de dislipidemias, percentual maior de adolescentes do sexo feminino apresentou níveis elevados de CT e LDLc. Por outro lado, a prevalência de níveis baixos de HDLc foi menor no sexo feminino. Não houve diferença por sexo na prevalência de hipertrigliceridemia.

Não foram observadas diferenças nos níveis médios dos lípides dos adolescentes mais jovens (12 a 14 anos) em relação aos mais velhos (15 a 17 anos). Da mesma forma, a prevalência de dislipidemias não foi diferente entre estes grupos.

A Figura 2 mostra as prevalências de dislipidemia pelas regiões do Brasil. Observa-se que para CT, LDLc e TG não houve diferenças entre as cinco regiões, com percentuais de alterações semelhantes. As prevalências de baixo HDLc foram elevadas nas regiões Norte e Nordeste.

**Figura 2:** Prevalência de dislipidemia por região geográfica. ERICA, Brasil, 2013-2014.

CT: colesterol total; HDLc: colesterol associado à lipoproteína de alta densidade; LDLc: colesterol associado à lipoproteína de baixa densidade; TG: triglicerídeos; N: Norte; NE: Nordeste; CO: Centro-Oeste; SE: Sudeste; S: Sul

## DISCUSSÃO

Embora medidas de prevenção primária de abrangência nacional pareçam estar reduzindo algumas formas de DCV no Brasil^21^, o impacto socioeconômico dessas doenças em nosso meio é significativo. O desenvolvimento de estratégias efetivas de prevenção cardiovascular depende do adequado reconhecimento dos fatores de risco no País. O ERICA é o maior estudo de prevalência de fatores de risco cardiovascular em adolescentes já realizado no Brasil. As prevalências de alterações lipídicas foram elevadas nos adolescentes brasileiros que estudam em municípios de mais de 100 mil habitantes, em especial as de níveis baixos de HDLc e de níveis elevados de CT.

Colesterol elevado correlaciona-se com o risco cardiovascular^11^ e, mesmo em crianças e adolescentes, está associado à presença de aterosclerose subclínica^12^ e ao risco de dislipidemia em idade adulta^20^. No ERICA, a definição de valores alterados seguiu as referências de uma diretriz nacional^28^, que difere de outras propostas, como o *National Cholesterol Education Program* (NCEP)^17^. Essas diferenças nas definições de dislipidemia devem ser consideradas ao se comparar os resultados do ERICA com outras populações. As comparações tornam-se ainda mais difíceis pela escassez de dados de base nacional na maior parte do mundo. Recentemente, a prevalência atual de dislipidemia em crianças e adolescentes nos Estados Unidos e a tendência temporal entre 1999 e 2012 foi publicada^10^. Cerca de 1/5 dos adolescentes americanos apresentam alguma alteração lipídica, e esse percentual decresceu no período avaliado. No ERICA, o mesmo percentual é encontrado apenas para o CT. Entretanto, parcela significativamente maior no Brasil apresenta níveis baixos de HDLc. Embora envolvendo população de etnia bastante distinta, um estudo de base nacional realizado na Coréia do Sul, mostrou valores médios de CT, LDLc, HDLc e TG semelhantes aos encontrados na nossa população^29^. No HELENA, estudo multicêntrico europeu com escolares da mesma faixa etária do ERICA, foram observados níveis de TG mais baixos do que os do ERICA (68 mg/dL [DP = 34]) para toda a população e padrão semelhante de distribuição por sexo: 63 mg/dL (DP = 31) no sexo masculino e 73 mg/dL (DP = 36) no sexo feminino^22^.

Já o estudo americano *National Health and Nutrition Examination Survey* III (NHANES III)(1988-1994) observou níveis de lípides superiores aos do ERICA para todos os parâmetros, CT 163 mg/dL (erro padrão [EP] = 1,0); LDLc 95 mg/dL (EP = 1,6); HDLc 49 mg/dL (EP = 0,4) e TG 93 mg/dL (EP = 2,4)^6^.

A abrangência e a representatividade nacional do estudo ERICA mostraram dados inéditos em nossa população. Aspectos distintos sobre faixa etária e sexo foram observados. Não há consenso se o ideal para definição de presença de dislipidemia em adolescentes é o uso de valores únicos de referência, como utilizado no ERICA e mais difusamente aplicado na rotina clínica, ou se o uso de curvas com valores específicos para sexo e idade.

Magnussen et al.^13^ compararam qual estratégia seria melhor para predizer a presença de dislipidemia na vida adulta, utilizando os valores de referência do NCEP^17^ e curvas para sexo e idade derivadas do NHANES^7^. Embora, a avaliação por curva tenha permitido melhor predição de níveis baixos de HDLc na vida adulta, todos os demais parâmetros lipídicos foram melhores preditos pelos valores de referência do NCEP, valores esses semelhantes aos usados no ERICA. No ERICA, idade não foi determinante de diferenças significativas. Os níveis médios de CT, LDLc, HDLc e TG não diferiram entre os adolescentes de 12-14 anos e 15-17 anos, corroborando a ideia de que valores únicos de referência para adolescentes possam ser utilizados para melhor identificação das alterações lipídicas^28^. Este dado reforça a necessidade do adequado conhecimento dos valores de referência aqui utilizados por aqueles que fazem o atendimento dessa população, o que hoje não ocorre^5^.

Nesta análise inicial, não foram desenvolvidas curvas específicas utilizando idade ano a ano, mas estudos que o fizeram mostram discreta redução de CT, LDLc e HDLc no início da adolescência de indivíduos do sexo masculino, com retorno aos níveis anteriores ao término da adolescência. Por outro lado, em adolescentes do sexo feminino, a idade parece ter relação direta e crescente com os níveis de LDLc^7^. Essa diferença entre os sexos ficou evidente no ERICA. Os níveis médios de CT, LDLc e TG foram mais elevados em adolescentes do sexo feminino, mas também os de HDLc. Consequentemente, a prevalência de níveis elevados de CT, LDLc e TG também foram maiores no sexo feminino, que apresentou menor prevalência de HDLc baixo.

Neste estudo, foram observadas diferenças regionais em relação à prevalência de dislipidemias. A prevalência de HDLc baixo foi significativamente maior nas regiões Norte e Nordeste do País. A prevalência de TG elevados também foi maior nessas duas regiões, mas observa-se superposição dos intervalos de confiança. Em Recife, região Nordeste, estudo realizado com escolares da rede pública também encontrou prevalência elevada de HDLc baixo (56,0% [IC95% 51,3-60,5])^19^. A combinação destas alterações metabólicas, HDLc baixo e TG elevados costumam coexistir, sobretudo na presença de obesidade^3^ e estilo de vida inadequado, e juntas são marcadoras da presença de moléculas de LDL menores e mais densas, mais pró-aterogênicas. Este padrão de dislipidemia, onde há pouca alteração dos níveis de LDL, mas predominando alterações de HDLc e TG, já foi descrito como padrão predominante na infância^9^. Dados recentes de aumento de mortalidade por doenças isquêmicas cardíacas nessas regiões^1^, sugerem que diferentes regiões do País podem estar em fases distintas no processo de transição epidemiológica, o que já foi bem descrito em diferentes países das Américas^14^. Análises futuras na mesma população permitirão melhor compreensão dos fatores associados a esse padrão de dislipidemia aterogênica observado nos adolescentes das regiões Norte e Nordeste do País.

Embora tenha sido a alteração lipídica menos frequente, o achado de 3,6% de prevalência de LDLc elevado (≥ 130 mg/dl) merece atenção especial. A presença de níveis elevados de LDLc em crianças e adolescentes deve ser o primeiro passo para o reconhecimento da hipercolesterolemia familiar, uma doença de causa genética e manifestação autossômica dominante, que acomete cerca de 1:500 indivíduos. No Brasil, estima-se que menos de 1,0% da população com hipercolesterolemia familiar esteja corretamente identificada^18^. Adolescentes com diagnóstico dessa doença estão expostos à alta carga lipídica desde o nascimento e precisam de acompanhamento especializado. Sem tratamento adequado, cerca de 50,0% dos homens irão apresentar um evento coronariano antes dos 50 anos^26^. Portanto, os dados do ERICA são uma oportunidade única de avaliação da real prevalência de hipercolesterolemia familiar no Brasil. A partir da identificação dos casos índex, uma estratégia de rastreamento em cascata poderá ser planejada.

Esta análise inicial dos dados lipídicos do ERICA teve como objetivo descrever a distribuição dos lípides e das dislipidemias em adolescentes escolares de municípios brasileiros com mais de 100 mil habitantes. Análises futuras poderão investigar fatores associados às dislipidemias, e suas combinações, tais como características de consumo alimentar, de hábitos de vida e histórico de morbidade familiar. Embora o ERICA seja um estudo seccional, por se tratar de população de adolescentes que ainda não apresenta complicações clínicas ou mortalidade decorrente das dislipidemias, é pouco provável que associações que venham a ser observadas sejam efeito de viés de sobrevida.

Em suma, as alterações lipídicas são frequentes nos adolescentes brasileiros. Intervenções de estilo de vida são fundamentais para melhorar esse panorama e geralmente são efetivas em curto prazo. Os dados sugerem que, ainda que estratégias de prevenção sejam planejadas nacionalmente, é fundamental que as diferenças regionais sejam reconhecidas para que ocorra adequada execução dessas estratégias.

## Footnotes

a Stata Corp. Stata: statistical software for professionals. Version 14.0. College Station, Texas; 2015.
